# Potential Benefits of Complementary Therapies for Women with Breast Cancer Undergoing Oncological Treatment: A Systematic Review

**DOI:** 10.3390/healthcare14111588

**Published:** 2026-06-04

**Authors:** María Dolores Guerra-Martín, Irene Delgado-Saldaña, Eleonora Magni, María Calderón-Fernández, Álvaro Borrallo-Riego

**Affiliations:** 1 Nursing Department, Faculty of Nursing, Physiotherapy and Podiatry, University of Seville, 41009 Seville, Spain; guema@us.es (M.D.G.-M.);; 2 Virgen del Rocío University Hospital, 41013 Seville, Spain; 3 Virgen de Valme University Hospital, 41014 Seville, Spain; 4 Red Cross University Nursing Centre, University of Seville, 41009 Seville, Spain

**Keywords:** complementary therapies, breast neoplasms, patient care, randomized controlled trials

## Abstract

**Highlights:**

**What are the main findings?**

- Complementary therapies such as acupuncture, mindfulness, massage, music therapy, and mind–body techniques showed potential benefits in improving pain, fatigue, anxiety, depression, and sleep quality in women with breast cancer undergoing chemotherapy or radiotherapy.
- The integration of complementary therapies demonstrated positive impacts across physical, psychological, and social dimensions, supporting a holistic approach to oncological care.

**What are the implications of the main findings?**

- Complementary therapies can be considered as safe adjuncts to conventional oncological treatment, potentially enhancing quality of life and overall well-being.
- The findings highlight the need for methodological standardization and long-term follow-up studies to consolidate evidence and optimize their clinical implementation.

**Abstract:**

**Background/Objectives:** Breast cancer is the most prevalent malignancy among women worldwide and remains a major cause of morbidity and mortality. Conventional treatments, while effective, often produce physical and psychological adverse effects that impair quality of life. Complementary therapies (CTs) have gained prominence as supportive strategies to mitigate these effects. This systematic review aimed to evaluate the potential benefits of CTs in women with breast cancer undergoing or having completed chemotherapy and radiotherapy. **Methods**: Following PRISMA 2020 guidelines and Cochrane standards, a systematic search was conducted in PubMed, CINAHL, Web of Science, and Scopus for studies published up to March 2026. Randomized controlled trials assessing CTs in breast cancer patients were included. Methodological quality was appraised using the PEDro scale, and risk of bias was evaluated using the Cochrane RoB 2 tool. **Results**: Thirty-two RCTs met the inclusion criteria. The interventions included acupuncture and related techniques, mindfulness-based therapies, massage, dance–movement therapy, music therapy, slow breathing, prayer, and natural products. Overall, CTs produced improvements in pain, fatigue, anxiety, depression, sleep quality, and social well-being, though the effects were sometimes therapy-specific and varied. **Conclusions**: Complementary therapies represent non-pharmacological strategies that may offer potential benefits across physical, emotional, and social outcomes in women with breast cancer. However, mixed results in certain therapies indicate that conclusions must be drawn cautiously. Future research should pursue methodological consistency and longer follow-ups.

## 1. Introduction

Cancer currently represents one of the leading causes of morbidity and mortality worldwide, accounting for approximately 10 million deaths in 2020. The most prevalent types include breast, lung, prostate, colorectal and rectal cancers, with around one-third of all cancer-related deaths being attributable to modifiable factors such as tobacco and alcohol consumption, excess body weight and physical inactivity [1]. The incidence of cancer has risen steadily in most countries, with a particularly high impact in low- and middle-income regions where mortality rates remain elevated [2]. In Spain, the Spanish Society of Medical Oncology [3] projects approximately 296,103 new cancer diagnoses, representing a 3.3% increase compared with the previous year, with breast cancer being the most frequent malignancy among women [4].

Breast cancer is the most prevalent neoplasm among women and remains one of the leading causes of cancer-related death worldwide [1]. It originates from the uncontrolled proliferation of mammary epithelial cells, which may invade adjacent tissues and metastasize to distant organs [5]. The disease exhibits significant biological and clinical heterogeneity, influenced by histological, molecular and hormonal characteristics [6]. Current therapeutic approaches include surgical interventions, chemotherapy, radiotherapy, hormone therapy (e.g., tamoxifen, aromatase inhibitors), and targeted therapies, with treatment selection depending on tumor stage, molecular profile and patient condition [7]. Despite advances in early detection and treatment, the physical, emotional and social sequelae of breast cancer continue to profoundly affect patients’ quality of life [8].

Oncological treatments, particularly chemotherapy and radiotherapy, are associated with a broad spectrum of adverse effects. Chemotherapy often causes alopecia, mucositis, nausea, fatigue, myelosuppression, peripheral neuropathy and cognitive impairment [9,10], while radiotherapy commonly results in acute dermatitis, pain, fatigue and esthetic alterations of the breast [11]. These physical effects frequently coexist with psychological manifestations such as anxiety, depression and body image disturbances [12], as well as social and occupational repercussions that increase patients’ vulnerability [13]. In this regard, complementary therapies play an essential role in therapeutic support and in the promotion of patients’ holistic well-being [14].

Over recent decades, the traditional biomedical model has evolved towards a holistic paradigm of care that acknowledges the interdependence of biological, psychological, social and spiritual dimensions of health [15]. This conceptual shift has fostered the integration of complementary therapies (CTs) into oncological care. CTs are defined as non-pharmacological interventions used alongside conventional medicine with the aim of alleviating symptoms, mitigating treatment-related adverse effects and enhancing quality of life [16,17]. Commonly used CTs include acupuncture, meditation, yoga, music therapy, hypnosis and cognitive-behavioral therapy [18]. Their use has increased globally: it is estimated that 81% of the Spanish population has used some form of CT, compared with 42% in the United States and 49% in France [19].

Current scientific evidence suggests that CTs can make a significant contribution to the comprehensive management of individuals with cancer by promoting relaxation, reducing anxiety and pain, improving mood, and fostering a sense of control over the disease. Nevertheless, the literature demonstrates considerable heterogeneity in reported outcomes, underscoring the need to update and synthesize the current body of evidence regarding their potential benefits [18,20,21]. While broader systematic reviews, including our own previous work (Guerra-Martín et al., 2021) [19], mapped the application of CTs across general cancer populations, they often clustered heterogeneous oncology patients together. The specific and novel contribution of the present review is to address this gap by focusing strictly on the unique clinical profiles, symptom burdens, and treatment stages specific to the breast cancer continuum, incorporating the latest clinical trials published up to March 2026.

Accordingly, the present study aims to update existing scientific knowledge on the potential benefits of complementary therapies in patients with breast cancer undergoing or having completed chemotherapy and radiotherapy, analyzing their impact on physical, psychological and social dimensions of patient well-being [18,20,21].

## 2. Materials and Methods

### 2.1. Study Design

A systematic review was conducted to identify, critically appraise, and synthesis the available scientific evidence on the potential benefits of CTs in women with breast cancer undergoing or having completed oncological treatment. Systematic reviews represent a secondary research design based on explicit, reproducible, and transparent methods aimed at minimizing bias and providing an integrated overview of findings derived from primary studies [22].

**Figure 1:**
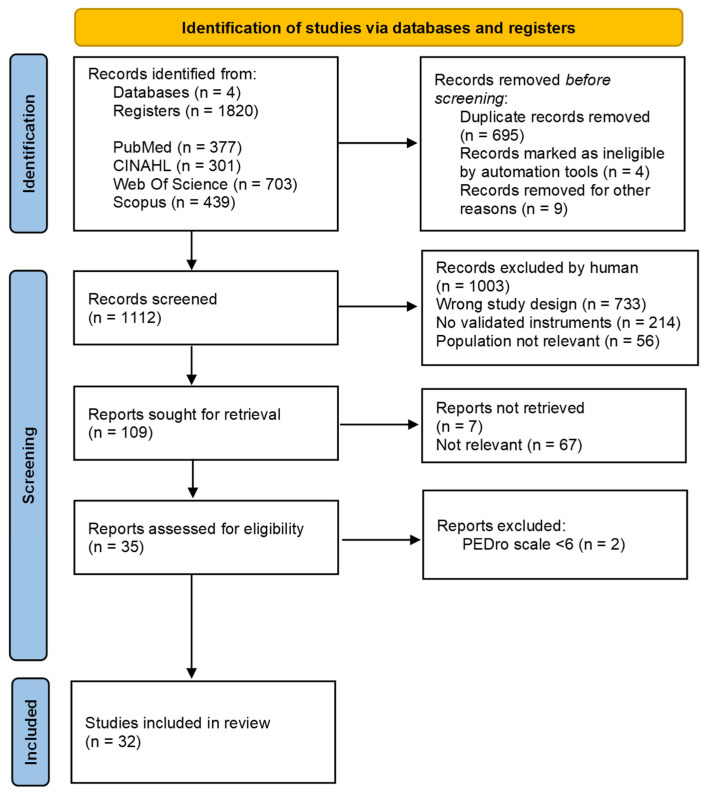
PRISMA flow diagram.

The methodological process adhered to the Cochrane Handbook for Systematic Reviews of Interventions and the Preferred Reporting Items for Systematic Reviews and Meta-Analyses (PRISMA 2020) guidelines (Figure 1) [23]. Two independent reviewers (E.M. and I.D.-S.) conducted the search, study selection, and data extraction in parallel, resolving discrepancies through consensus or the intervention of a third reviewer (M.D.G.-M.).

### 2.2. Protocol Registration

The methodological protocol was registered in PROSPERO under the identifier CRD420251008594.

### 2.3. Information Sources and Search Strategy

The literature search was conducted up to March 2026 across the international databases PubMed, CINAHL, Web of Science (WOS), and Scopus. Controlled vocabulary terms from Medical Subject Headings (MeSH) were employed, combined using the Boolean operators AND and OR. The search strategy applied across all databases was as follows: ((“Breast Neoplasms” OR “Breast Tumor” OR “Cancer of Breast” OR “Breast Carcinoma”) AND (“Complementary Therapies” OR “Alternative Medicine” OR “Alternative Therapies” OR “Complementary Medicine”) AND (“Drug Therapy” OR “Chemoradiotherapy” OR “Chemotherapy” OR “Radiotherapy” OR “Radiation Therapy”)). No language or time restrictions applied.

### 2.4. Eligibility Criteria

The inclusion criteria were: (1) randomized controlled trials (RCTs); (2) studies assessing complementary therapies administered to patients with breast cancer; (3) publications dated between 2015 and March 2026; and (4) a methodological quality score ≥6 on the PEDro scale [24]. Although blinding is a standard quality indicator, the absence of blinding was not an exclusion criterion, given the inherent impossibility of blinding participants in certain manual and mind–body interventions.

### 2.5. Study Selection and Data Extraction

Record management and screening were performed using the electronic platform Covidence Extraction 1. The selection process comprised three sequential phases: 1. Initial screening by title and abstract to remove duplicates and irrelevant studies. 2. Full-text assessment to confirm eligibility against predefined inclusion criteria. 3. Methodological appraisal using the PEDro scale, with the exclusion of studies scoring below six.

Data extraction followed a standardized and internally validated protocol. The following information was retrieved from each included study: Authors, cancer stages, treatment, sample, country, measured variables, measurement instrument, assessment time points, CTs applied, number of sessions, duration and their potential benefits. A narrative synthesis was performed, grouping the findings according to the nature of the CTs and its impact on physical, psychological, and social outcomes.

### 2.6. Methodological Quality and Risk-of-Bias Assessment

The methodological quality of included studies was appraised using the PEDro scale [25], comprising 11 items that evaluate internal validity, external validity, and reproducibility. Studies scoring 9–10 were classified as having very high quality, 6–8 as good, 5–6 as fair, and below 4 as poor.

The risk of bias was assessed using the Revised Cochrane Risk-of-Bias Tool for Randomized Trials (RoB 2) [26]. This instrument examines five key domains: random sequence generation, deviations from intended interventions, incomplete outcome data, measurement bias, and selective reporting. Studies were categorized as low risk, some concerns, or high risk [27].

Assessment of reporting biases, such as publication bias, was not performed via funnel plots or formal statistical tests due to the substantial clinical and methodological heterogeneity of the included interventions and the qualitative nature of the synthesis. Furthermore, while the certainty of evidence was considered during the discussion of findings, a formal GRADE (Grading of Recommendations Assessment, Development and Evaluation) assessment was not conducted as it was not part of the initial study protocol.

## 3. Results

### 3.1. Study Selection and Methodological Quality

A total of 1820 studies were identified, of which 32 met the inclusion criteria. Methodological quality was assessed using the PEDro scale (Table S1); all included studies reached the minimum score of 6/10. Specifically, two studies achieved a maximum score of 10/10 [28,29], five scored 9/10 [30,31,32,33,34], fourteen scored 8/10 [35,36,37,38,39,40,41,42,43,44,45,46,47,48], seven scored 7/10 [49,50,51,52,53,54,55], and four scored 6/10 [56,57,58,59]. Three studies were excluded because they scored less than 6 on the PEDro scale [44,60,61].

Risk of bias was assessed using the RoB 2.0 tool (Figures S1 and S2). Overall, a high risk of bias was observed in the “deviations from intended interventions” domain across most studies due to the impossibility of blinding participants in exercise or manual therapy trials. However, a low risk of bias was observed across most other domains, particularly in the reporting of results.

### 3.2. Characteristics of the Studies

The 32 included studies were all randomized clinical trials, with participants exclusively women, having a mean age between 48 and 66 years (Table 1).

Regarding blinding, thirteen studies were reported as single-blind [34,35,37,39,41,42,43,47,49,50,52,53,59], ten were double-blind [28,29,30,31,32,33,38,40,46,48], and nine did not specify the type of blinding or were unblinded due to the nature of the intervention [36,45,51,54,55,56,57,58,62].

The studies included patients with various cancer stages, ranging from 0 to III. Twelve studies included stages I–III [28,35,45,47,48,49,50,53,54,62]. Four studies included stages 0–III [37,40,52,56]. In thirteen studies, the exact cancer stage was not reported.

### 3.3. Instruments and Measurement Scales

A wide range of validated instruments were used across the included studies to assess fatigue, psychological distress, pain, sleep quality, and well-being (Table 2).

Fatigue was primarily assessed using the Brief Fatigue Inventory (BFI) [28,54,62]. Other tools included the Multidimensional Fatigue Inventory (MFI-20) [35,52], the Piper Fatigue Scale (PFS) [36,39], the PROMIS fatigue scale, the QuickPIPER [45], and the PFS [48].

Anxiety and depression were most frequently measured using the Hospital Anxiety and Depression Scale (HADS) [28,32,58], the State-Trait Anxiety Inventory (STAI) [36,49], the Profile of Mood States (POMS) [45], and physiological measures such as EDA and eye-blink probability [33].

Pain was assessed using the Brief Pain Inventory (BPI) [20,24], the Numeric Rating Scale (NRS) [38,39], and specific tools for neuropathic pain such as the NTSS-6 [31], S-LANSS [39] and the Visual Analog Scale (VAS) [29,45,47].

Cognitive function was objectively measured using the Auditory Sustained Attention Test (ASAT) [33].

Quality of life and well-being were assessed with the FACT-B/FACT-G [30,41,50] and the EORTC QLQ-C30 [28,43,57].

Other domains included sleep quality, measured almost exclusively with the Pittsburgh Sleep Quality Index (PSQI) [40,54,59].

### 3.4. Complementary Therapies Applied

The CTs investigated across the included studies are summarized in Table 2.

Acupuncture and related techniques were a major focus, including therapeutic acupuncture, electroacupuncture, auricular/self-acupressure, auricular acupressure, acupressure at the Neiguan (PC6) point, and infrared laser moxibustion [28,29,46,47,50,58,59].

Mind–body therapies were integrated alongside mindfulness, slow breathing, Qigong/Tai Chi, Baduanjin, and HUE techniques [40,41,48,54,56,58].

Natural products such as Peppermint extract [29] and homeopathic medicinal products [33] were both applied in double-blind studies.

Other CTs included music therapy [36,38], natural products such as ginseng and mistletoe [30,57], massage therapy (classical and Swedish) [39,52], dance–movement therapy [37], neuromuscular taping (NMT) [45], and daily prayer [32].

### 3.5. Potential Benefits of Complementary Therapies

It is important to note that most primary studies relied on statistical significance rather than standardized effect sizes, limiting uniform comparison of effect magnitudes. To facilitate the interpretation of the key findings, Table 3 summarizes the clinical effects categorized by therapy type.

#### 3.5.1. Physical Outcomes

1. Pain: Seven studies demonstrated significant pain reduction through CTs such as classical massage, auricular acupressure, dance–movement therapy, music therapy, moxibustion, neuromuscular taping, and mistletoe injections [28,31,38,39,45,57].
2. Fatigue: Improvements in fatigue were significantly reported in studies involving mindfulness, Swedish massage, neuromuscular taping, Shenqi Fuzheng granules, Qigong/Tai Chi, laser moxibustion, and acupressure [28,40,41,45,48,59].
3. Lymphedema: Acupuncture improved subjective symptoms of heaviness and tightness but did not significantly reduce arm volume [50]. Manual lymphatic drainage showed no additional benefit over standard care regarding arm volume reduction [43].
4. Nausea and Vomiting: Peppermint extract [29] and acupressure [47] significantly reduced chemotherapy-induced nausea and vomiting (CINV).

#### 3.5.2. Psychological Outcomes

1. Stress: Significant stress reduction followed mindfulness, dance–movement therapy, Bali Yoga, peppermint extract, and daily prayer [29,32,37,49,56].
2. Anxiety and Depression: Improvements were reported with mindfulness, Qigong, prayer, slow breathing, and acupuncture [36,49,54,58]. Music intervention specifically improved anxiety and quality of life but not fatigue [36].
3. Sleep: Interventions such as electroacupuncture, self-acupressure, Shenqi Fuzheng granules, Baduanjin, and mindfulness [40,48,54,59,62] yielded positive outcomes for sleep disturbances.
4. Cognition: Homeopathy [33] showed a significant recovery of attention performance 1-month post-radiotherapy.

#### 3.5.3. Social Outcomes

Ginseng supplementation, HUE techniques, neuromuscular taping, ginseng, and slow breathing [30,42,45,54] demonstrated improvements in social functioning and overall quality of life.

## 4. Discussion

Regarding the methodological characteristics of the trials included in this review, all exclusively involved women, which is consistent with previous reviews focusing on female populations [63]. The sample sizes in this updated selection ranged from 21 to 424 participants, with larger trials such as Zick et al. [59] providing higher statistical power than typical pilot studies [64]. The mean age of participants was between 48 and 66.3 years [35,54], aligning with data from Uceda-Escobar et al. [65]. All 32 studies were randomized clinical trials (RCTs), providing a robust level of evidence [66] according to methodological standards [67,68]. However, according to Table 1, while thirteen studies were single-blind and ten were double-blind, nine did not specify the type of blinding or were unblinded. This lack of blinding, often inherent to physical interventions like yoga or massage, may introduce performance bias [22].

In terms of assessment instruments, the studies employed standardized tools. Fatigue was primarily assessed using the BFI [28,62] and the MFI-20 [35], the QuickPIPER [45], and the Piper Fatigue Scale (PFS) [48] were widely used in clinical contexts [69]. Anxiety and depression were measured with the HADS [32,58] and the STAI [36], and the Profile of Mood States (POMS) [45], consistent with Robles-Buelvas et al. [70]. Quality of life was most frequently assessed using the FACT-B/FACT-G [30,48,50,55] and the EORTC QLQ-C30 [43,45]. Notably, the NTSS-6 was used for neuropathic pain [31], the Visual Analog Scale (VAS) for general and nausea-related pain [29,38], and the PSQI for sleep quality across multiple interventions [48,54,59]. Furthermore, physiological indicators like Electro-Dermal Activity (EDA) and eye-blink probability were utilized to assess stress levels [33].

Concerning the complementary therapies (CTs) applied, acupuncture and related techniques (acupressure, moxibustion) confirmed their efficacy in reducing fatigue and pain [28,59]. Newer modalities such as neuromuscular taping (NMT) demonstrated potential benefits for aromatase inhibitor-associated arthralgia [45], while foot reflexology and peppermint extract [29] addressed chemotherapy-induced fatigue and nausea, respectively. These results align with neuroimmunological modulation mechanisms [71,72]. Mind–body therapies, including mindfulness, Qigong, and Tai Chi [41,56,58], consistently improved stress and depression. Specifically, Qigong demonstrated significant benefits for cognitive function [41]. Additionally, homeopathic medicinal products (HMP) showed a significant recovery of attention performance in patients following radiotherapy [33]. Music therapy effectively reduced anxiety and improved quality of life [36,38]. Regarding natural products, ginseng showed significant improvements in all quality-of-life subscales [30], while Shenqi Fuzheng granules (SQFZ) improved sleep and fatigue [48] and mistletoe injections improved pain and neutropenia [57]. Massage therapy remained effective for pain and fatigue [39,52] and spiritual practices like daily prayer supported spiritual well-being [32].

The physical potential benefits of CTs was evidenced by reductions in pain and fatigue. Interventions such as classical massage, music, and auricular acupressure produced clinically meaningful improvements [31,37,38,39]. Significant results were also found for chemotherapy-induced nausea and vomiting (CINV) through acupressure wristbands [47] and oral peppermint extract [29]. However, results for lymphedema were more nuanced; while acupuncture improved subjective symptoms like heaviness, it did not significantly reduce arm volume [50]. Similarly, manual lymphatic drainage did not provide additional volume reduction over standard care [43]. Regarding fatigue, 12 trials demonstrated significant reductions [28,40,59].

In the psychological dimension, CTs were effective in reducing stress, anxiety, and depression. Mindfulness, dance, and prayer reduced stress levels [32,37,56]. Depression and anxiety improved following Bali Yoga, Qigong, and slow-breathing exercises [35,54,58]. The use of peppermint extract also showed a significant reduction in anxiety scores during chemotherapy [29]. Regarding sleep, acupuncture, foot reflexology, and SQFZ granules mindfulness, and Baduanjin demonstrated significant benefits [40,48,54,62].

CTs also positively influenced the social dimension of well-being. Daily breathing, energy-based techniques, and meditation practices enhanced social functioning and perceived support [30,42,54]. Neuromuscular taping further improved global health status and social functioning subscales [45].

Comparing these findings with broader umbrella reviews, such as our previous work (Guerra-Martín et al., 2021) [19], the present study corroborates the general safety and multidimensional benefits of CTs. However, it is noteworthy that substantial clinical and methodological heterogeneity persists even when strictly isolating the breast cancer population. This enduring heterogeneity is largely driven by the broad spectrum of disease stages (0-III) included, the inherently different baseline symptom burdens between patients in active treatment versus survivorship phases, and the wide variation in CT protocols (e.g., differing session frequencies, durations, and delivery methods). Furthermore, it must be explicitly acknowledged that conducting a quantitative meta-analysis was not feasible due to this substantial clinical and methodological heterogeneity.

Among the limitations of this systematic review are the methodological heterogeneity regarding cancer stages (ranging from stage 0 to III) and the diversity of treatment contexts (active chemotherapy vs. survivorship), which hinders direct comparability. Furthermore, the inherent difficulty of blinding in mind–body and manual therapies remains a challenge for achieving “Low Risk” ratings in all RoB 2 domains. However, the inclusion of pharmacological and natural product trials [29,48] provided studies with higher methodological scores (PEDro ≥8).

The 32 included studies, all clinical trials, provide a broad overview of complementary therapies in female breast cancer patients. A significant methodological limitation observed is the high risk of bias in the ‘deviations from intended interventions’ domain. This is particularly pervasive in mind–body and manual therapies where participant blinding is largely unfeasible. This risk necessitates cautious interpretation, as placebo effects or performance biases cannot be entirely ruled out.

Furthermore, although a formal GRADE assessment was not conducted as it was not predefined in our protocol, the omission of this evaluation limits the strength of clinical recommendations that can be drawn. Consequently, the findings presented herein are framed as identifying “potential benefits” rather than establishing definitive clinical “effectiveness”.

The inclusion of patients across various cancer stages (0 to III) and distinct treatment phases (active chemotherapy versus survivorship) introduces clinical heterogeneity. While these populations possess fundamentally different symptom burdens, they were pooled narratively in this review to provide a comprehensive map of how CTs are applied across the entire continuum of breast cancer care.

Finally, while the heterogeneity precluded the use of formal funnel plot analyses, a narrative assessment of publication bias—such as observing the lack of published negative trials in certain domains—suggests a likelihood of publication bias that should be rigorously tested in future quantitative meta-analyses.

## 5. Conclusions

This systematic review underscores the increasing evidence supporting the role of CTs in women with breast cancer, now encompassing 32 randomized clinical trials with participants aged between 48 and 66.3 years. Despite methodological heterogeneity in the cancer stage (0–III) and treatment phase (active chemotherapy, radiotherapy, or survivorship), the collective findings reveal the global relevance of integrative oncology.

A broad range of validated tools was used to assess physical, psychological, and social outcomes, reflecting the multidimensional impact of these interventions. In addition to standard scales like the BFI and HADS, this update highlights the use of specialized tools for neuropathic pain (NTSS-6), shoulder disability (SPADI), and objective cognitive assessment (ASAT). Acupuncture and related techniques, mindfulness-based practices, Qi Gong/Tai Chi, and music therapy were the most frequently investigated, complemented by emerging modalities such as neuromuscular taping, foot reflexology, homeopathic medicinal products, and phytotherapy (peppermint extract and Shenqi Fuzheng granules).

Overall, complementary therapies demonstrated significant benefits in reducing pain, fatigue, anxiety, depression, and sleep disturbances. Notably, this review provides new evidence for the potential benefits of CTs in managing chemotherapy-induced nausea and vomiting (CINV) and radiation-induced cognitive dysregulation. These results support their integration as effective adjuncts to conventional treatment, promoting a holistic approach to cancer care. While studies involving natural products and homeopathy achieved higher methodological quality scores (PEDro ≥8), the inherent challenge of blinding in manual and mind–body therapies remains. Future studies should aim for methodological standardization and long-term follow-up to consolidate the evidence and optimize their clinical application in breast cancer management.

This systematic review highlights the growing body of evidence supporting the role of complementary therapies in women with breast cancer. Overall, interventions such as acupuncture, mindfulness, and specific natural products may offer potential benefits in reducing pain, fatigue, anxiety, and sleep disturbances. However, conclusions must accurately reflect the nuanced and therapy-specific nature of the evidence, as findings are sometimes mixed (e.g., Bali Yoga showing no significant effect on fatigue or nausea, and manual lymphatic drainage providing no additional benefit over standard care). Future studies must prioritize methodological standardization, strict reporting of effect sizes, and long-term follow-ups to consolidate these findings.

## Figures and Tables

**Table 1:** Characteristics of studies.

Author and Year	Blinding	Cancer Stages/Treatment	Sample/Country
Anestin et al., 2017 [49]	Single-blind	Stages I–III/Chemotherapy	N = 82 (52 IG, 30 CG), 100% women/Canada
Anestin et al., 2022 [35]	Single-blind	Stages I–III/Chemotherapy	N = 48 (25 IG, 23 CG), 100% women/Canada
Bao et al., 2018 [50]	Single-blind	Stages I–III/Treatment completed, Surgery/radiotherapy/chemotherapy	N = 82 (40 IG, 42 CG), 100% women/USA
Bower et al., 2015 [56]	Not specified	Stages 0–III/Treatment completed, unspecified	N = 71 (39 IG, 32 CG), 100% women/USA
Brinkhaus et al., 2019 [55]	Not specified	Newly diagnosed (stage not specified)/Active treatment: Chemotherapy	N = 150 (75 IG, 75 CG), 100% women/Germany
Chuang & Chen, 2014 [36]	Not specified	Stage not specified/Treatment completed, Mastectomy	N = 170 (Not specified), 100% women/Taiwan
Cohen et al., 2021 [51]	Not specified	Stage not specified/Survivors/Treatment complete	N = 40 (Not specified), 100% women/ USA
Conejo et al., 2018 [45]	Not specified	Stages I–IIIA/Surgery, chemotherapy, radiotherapy) + adjuvant endocrine therapy	N = 40 (20 IG, 20 CG), 100% women/Spain
Dolev et al., 2021 [33]	Double-blind	Unilateral BC (stage not specified)/Radiotherapy post-chemotherapy	N = 57 (38 IG, 19 PG), 100% women/Israel
Eng et al., 2025 [46]	Double-blind	Stage not specified/Chemotherapy	N = 51 (26 IG, 25 CG), 100% women/South Korea
Hamidian et al., 2023 [30]	Double-blind	Stages II–III/Chemotherapy	N = 40 (15 IG, 25 CG), 100% women/Iran
Ho et al., 2016 [37]	Single-blind	Stages 0–III/Adjuvant radiotherapy	N = 139 (69 IG, 70 CG), 100% women/China
Hsieh et al., 2019 [38]	Double-blind	Stage not specified/Chemotherapy	N = 60 (30 IG, 30 CG), 100% women/Taiwan
Izgu et al., 2019 [39]	Single-blind	Stage not specified/Chemotherapy	N = 40 (20 IG, 20 CG), 100% women/Turkey
Jafarimanesh et al., 2020 [29]	Double-blind	Stage not specified/Chemotherapy	N = 84 (42 IG, 42 CG), 100% women/Iran
Jung et al., 2025 [31]	Double-blind	Stage not specified/Chemotherapy	N = 51 (26 IG, 25 CG), 100% women/South Korea
Kinkead et al., 2017 [52]	Single-blind	Stages 0–III/Surgery + radiotherapy/chemotherapy	N = 66 (Not specified), 100% women/USA
Larkey et al., 2014 [40]	Double-blind	Stages 0–III/Treatment completed, Surgery/radiotherapy/chemotherapy	N = 87 (42 IG, 45 CG), 100% women/USA
Mao et al., 2024 [28]	Double-blind	Stages I–III/Treatment completed, Surgery + radiotherapy/chemotherapy	N = 140 (56 IG, 56 PG, 28 CG), 100% women/China
Miranda et al., 2020 [32]	Double-blind	Stage not specified/Adjuvant radiotherapy	N = 31 (16 IG, 15 CG), 100% women/ Brazil
Myers et al., 2019 [41]	Single-blind	Stages I–III/Treatment completed, Chemotherapy (+radiotherapy), endocrine therapy was allowed	N = 50 (19 IG1, 20 IG2, 11 CG-Support Group), 100% women/USA
Nguyen et al., 2018 [42]	Single-blind	Stage not specified/External beam radiotherapy	N = 32 (16 IG, 16 CG), 100% women/Australia
Pelzer & Tröger, 2018 [57]	Not specified	Stage not specified/Adjuvant radiotherapy	N = 95 (Not specified), 100% women/Serbia and Germany
Serra et al., 2023 [53]	Single-blind	Stages I–III/Endocrine therapy post-surgery-radiotherapy	N = 47 (27 IG, 20 CG), 100% women/USA
Shao et al., 2026 [48]	Double-blind	Stages I–III/Chemotherapy	N = 100 (50 IG, 50 CG), 100% women/China
Tambour et al., 2018 [43]	Single-blind	Lymphoedema Stages II–III/Treatment completed, Chemotherapy/radiotherapy	N = 77 (39 IG, 38 CG), 100% women/Denmark
Tsai et al., 2021 [47]	Single-blind	Stages I–III/Chemotherapy	N = 104 (52 IG, 52 CG), 100% women/Taiwan
Wang et al., 2024 [54]	Not specified	Stages I–IIIa/Treatment completed, unspecified	N = 72 (36 IG, 36 CG), 100% women/China and Australia
Yun et al., 2017 [58]	Not specified	Stages I–III/Treatment completed, Surgery + adjuvant chemotherapy	N = 91 (46 IG, 45 CG), 100% women/South Korea
Zhang et al., 2020 [62]	Not specified	Stages I–III/Chemotherapy	N = 100 (50 IG, 50 CG), 100% women/China
Zhu et al., 2018 [34]	Single-blind	Stage not specified/Chemotherapy	N = 80 (40 IG, 40 CG), 100% women/Iran
Zick et al., 2016 [59]	Single-blind	Stages 0–III/Treatment completed, Chemotherapy/radiotherapy	N = 424 (141 IG1, 142 IG2, 141 CG), 100% women/USA
The Authors.			

IG: intervention group; PG: placebo group; CG: control group. Note: The full Table 1 content is maintained with the 32 studies. Thirteen studies did not clearly specify the tumor stage.

**Table 2:** Instruments and measurement scales. Complementary therapies applied and their potential benefits.

Author and Year	Measured Variables/Measurement instrument/Assessment Time Points	Complementary Therapy Applied/Number of Sessions/Duration	Potential Benefits of the Interventions/Context
Anestin et al., 2017 [49]	**Variables**: Nausea, vomiting (CINV), anxiety. **Instruments**: MANE, STAI. **Assessment**: First cycle, midcourse, and eighth week.	**Therapy**: Bali Yoga Program vs. Waitlist. **Sessions**: 8 sessions. **Duration**: 90 min weekly + 20–40 min daily home practice.	The IG showed no significant difference for nausea/vomiting compared to control, but a significant decrease in anxiety. Context: N = 82 randomized; attrition bias observed as non-completers had significantly more pre-existing muscle pain.
Anestin et al., 2022 [35]	**Variables**: Cancer-related fatigue (CRF). **Instruments**: MFI-20. **Assessment**: Baseline, 8 weeks, and 16 weeks.	**Therapy**: Bali Yoga Program vs. Waitlist. **Sessions**: 8 sessions. **Duration**: 90 min weekly + 20–40 min daily home practice.	The IG showed no significant main effect on general or mental fatigue, but significant improvement in motivation. Context: N = 48 randomized; significant attrition bias noted with non-completers suffering from baseline muscle pain.
Bao et al., 2018 [50]	**Variables**: Lymphedema, bioimpedance. **Instruments**: Tape measure, bioimpedance. **Assessment**: Baseline, 6 weeks, and 3 months post-treatment.	**Therapy**: Manual acupuncture vs. Waitlist. **Sessions**: 12 sessions. **Duration**: Twice weekly for 6 weeks.	Did not find evidence that acupuncture reduced lymphedema or bioimpedance compared to waitlist control. Context: N = 82 randomized; minor adverse events reported (e.g., 58% bruising), high retention.
Bower et al., 2015 [56]	**Variables**: Stress, depression, sleep, inflammation, fatigue, hot flashes. **Instruments**: PSS, CES-D, FSI, PSQI, BCPT, biomarkers. **Assessment**: Baseline, post-intervention, and 3-month follow-up.	**Therapy**: Mindful Awareness Practices (MAPS) vs. Waitlist. **Sessions**: 6 sessions. **Duration**: 120 min weekly + 5–20 min daily home practice.	The IG showed significant reductions in stress, proinflammatory signaling, fatigue, sleep disturbance, and hot flashes. Context: N = 71 younger survivors randomized; excellent adherence and follow-up rate.
Brinkhaus et al., 2019 [55]	**Variables**: Disease-specific quality of life, chemotherapy-induced side effects (nausea, pain, fatigue). **Instruments**: FACT-B (Primary), VCS (Vomiting), NRS (Pain). **Assessment**: Baseline, 3 months, and 6 months (end of intervention).	**Therapy**: Prophylactic acupuncture (individualized) plus standard care vs. Standard care alone. **Sessions**: 10 to 15 sessions. **Duration**: Over a 6-month period (during chemotherapy).	The IG showed no statistically significant difference in the primary outcome (FACT-B total score) at 6 months compared to the CG. However, qualitative interviews revealed high patient satisfaction and a subjective sense of improved well-being and coping. Context: Randomized pragmatic trial; attrition was noted (106 participants evaluable at 6 months).
Chuang & Chen, 2014 [36]	**Variables**: QoL, anxiety, fatigue. **Instruments**: EORTC QLQ-C30, STAI, PFS. **Assessment**: Baseline, 4, 8, and 12 weeks.	**Therapy**: Music intervention vs. Control. **Sessions**: 12 sessions. **Duration**: 60 min weekly.	The IG showed significant improvements in QoL and anxiety compared to the CG, but no significant differences in fatigue. Context: N = 170 mastectomy patients; greater quality of life observed prominently at 12 weeks.
Cohen et al., 2021 [51]	**Variables**: Fatigue, anxiety, depression, tiredness/energy. **Instruments**: Piper Fatigue Scale, AD ACL, HADS. **Assessment**: Baseline and 3 sessions over 7 days.	**Therapy**: Aerobic exercise + technology-guided MBSR vs. Aerobic exercise vs. Relaxation. **Sessions**: 3 sessions. **Duration**: 40 min active (20 min exercise + 20 min MBSR) in a 90 min window.	The combined group achieved a large reduction in fatigue (d = 0.91) and increased energy compared to aerobic exercise alone. Context: N = 40 randomized; 100% adherence and retention rates, highly acceptable and feasible.
Conejo et al., 2018 [45]	**Variables**: Subjective pain, quality of life (QoL), fatigue, mood states, grip strength, pressure pain threshold (PPT), and plasma proteins (CRP, CK). **Instruments**: VAS (Primary), EORTC QLQ-C30, POMS, QuickPIPER, SFI, ULFI, BADIX, pressure algometry, and dynamometry. **Assessment**: Baseline (T1), 1 week (T2), and 5 weeks (T3)	**Therapy**: Real Neuromuscular Taping (NMT) applied to painful areas (cervical, lumbar, or wrist) vs. Sham NMT (shorter, narrower strips applied in neutral position). **Sessions**: 3 intervention sessions. **Duration**: 5 weeks total, with tapes maintained for 7 days per session.	The experimental group showed significant improvements in subjective pain (VAS, *p* = 0.009), global health status/QoL (*p* = 0.005), fatigue (*p* = 0.01), and pain symptoms (*p* = 0.04) at 5 weeks compared to the control. No significant differences were found for grip strength, pressure pain threshold, or plasma proteins. Context: Pilot pragmatic trial; high adherence with only 10% total attrition.
Dolev et al., 2021 [33]	**Variables**: Attention (ASAT), Anxiety (EDA, Eye-blink)/Baseline, 2, 4, 8 weeks. **Instruments**: Homeopathy (HMP) / Daily granules / 8 weeks. **Assessment**: Sig. recovery of attention performance (*p* < 0.035) in IG at 1 month post-RT	**Therapy**: Routine Homeopathic Medicinal Product (HMP) complex (including Cadmium sulphuratum, Phosphoricum acidum, Radium Bromide, X-ray, and Carcinosinum burnett) vs. Placebo (carrier sucrose granules). **Sessions**: Daily oral administration of granules. **Duration**: Throughout the 5-week radiotherapy period and continuing until the 8-week follow-up.	The intervention group (CAM) showed a significant recovery of attention performance (*p* < 0.035) and preserved baseline levels of eye-blink probability (lower fatigue/anxiety) compared to the placebo group, which showed deterioration in attention 1 month after radiation. No significant differences were found between groups for startle intensity or skin conductance. **Context:** Randomized double-blind placebo-controlled trial; 18.5% total attrition primarily due to hearing deterioration or lack of motivation.
Eng et al., 2025 [46]	**Variables**: Neuropathic pain intensity, CIPN-related symptoms, and interference with daily activities. **Instruments**: Visual Analog Scale (VAS) for pain, Total Neuropathy Score (TNS), and the EORTC QLQ-CIPN20. **Assessment**: Baseline, Week 1, Week 2, Week 3 (end of intervention), and Week 7 (1-month follow-up).	**Therapy**: Auricular Acupressure (AA) using Vaccaria seeds on specific points (Shenmen, Finger, Hand, Toe, Foot) vs. Sham AA (non-adhesive seeds on non-acupoints). **Sessions**: 3 cycles of seed replacement. **Duration**: 3 weeks of active treatment (seeds kept for 5 days per week, with pressure applied 3 times daily for 2 min).	The intervention group showed a significant reduction in neuropathic pain (VAS) and CIPN symptoms (QLQ-CIPN20) at the 3rd week and 7th week compared to the sham group. However, the objective Total Neuropathy Score (TNS) did not show a significant difference between groups. Context: Randomized double-blind sham-controlled trial; 13.7% attrition rate.
Hamidian et al., 2023 [30]	**Variables**: Quality of life. **Instruments**: FACT-B. **Assessment**: Baseline, 2 weeks after 2nd cycle, and 2 weeks after 4th cycle.	**Therapy**: Panax Ginseng vs. Placebo. **Sessions**: Daily. **Duration**: 1 g/day concurrent with chemotherapy.	The IG showed significant improvements in physical, social, emotional, and functional well-being versus a declining trend in the placebo group. Context: N = 41 randomized; double-blind design with only 1 dropout.
Ho et al., 2016 [37]	**Variables**: Stress, pain, fatigue, QoL, anxiety, depression, sleep. **Instruments**: PSS, HADS, BFI, BPI, PSQI, FACT-B. **Assessment**: Baseline and after 3 weeks.	**Therapy**: Dance–movement therapy vs. Waitlist. **Sessions**: 6 sessions. **Duration**: 90 min each (twice weekly).	The IG showed significant improvements in stress and pain intensity, but no improvements in anxiety, depression, fatigue, sleep, or QoL. Context: N = 139 randomized; low attrition and intent-to-treat analysis used.
Hsieh et al., 2019 [38]	**Variables**: Symptom severity, pain, fatigue. **Instruments**: TRSC-Chinese, NRS-101, MFSI-SF-C. **Assessment**: Weeks 0, 6, 12, and 24.	**Therapy**: Home-based music (MP3) vs. Ambient music. **Sessions**: 5 times per week. **Duration**: 30 min daily for 24 weeks.	Reduction in overall symptoms, pain, and multidimensional fatigue, which persisted in the long term. Context: N = 60 randomized; double-blind parallel-group RCT, 16% attrition rate.
Izgu et al., 2019 [39]	**Variables**: Neuropathy, QoL, nerve conduction. **Instruments**: S-LANSS, EORTC QLQ-CIPN20, NCS. **Assessment**: Weeks 0, 4, 8, 12, and 16.	**Therapy**: Classical massage vs. Control. **Sessions**: 12 sessions. **Duration**: 30 min before chemo (20 min feet, 10 min hands).	Significant reduction in peripheral neuropathic pain and improved sensory/motor QoL, with positive effects on nerve conduction studies. Context: N = 40 randomized; assessor-blinded trial with no dropouts reported.
Jafarimanesh et al., 2020 [29]	**Variables**: Severity of nausea, vomiting, and anorexia. **Instruments**: Visual Analog Scale (VAS) for nausea and anorexia severity; frequency of vomiting episodes was recorded as a numerical count. **Assessment**: Before chemotherapy (baseline), immediately after, 24 h after, and 48 h after chemotherapy.	**Therapy**: Peppermint (*Mentha piperita*) extract (40 drops mixed in 20 cc of water) vs. Placebo (40 drops of distilled water in 20 cc of water). Frequency: Administered orally every 8 h. **Duration**: During the first 48 h following the chemotherapy cycle.	The intervention group showed a significant reduction in the severity of nausea and vomiting at 24 and 48 h post-chemotherapy compared to the control group (*p* < 0.05). No significant differences were observed between groups regarding the severity of anorexia. Context: Randomized double-blind placebo-controlled trial; all participants completed the 48 h follow-up with no dropouts reported.
Jung et al., 2025 [31]	**Variables**: Neuropathic pain, CIPN symptoms. **Instruments**: TNS, NRS, CIPN-20. **Assessment**: Baseline, weeks 1, 2, 3, and 7 (1-month follow-up).	**Therapy**: Auricular acupressure vs. Sham. **Sessions**: 3 seed replacements. **Duration**: 5 days on, 2 days off per week; 2 min pressure 3 per day.	Significant reduction in neuropathic pain and CIPN symptoms; Total Neuropathy Score (TNS) showed no significant group difference. Context: N = 51 randomized; dropout rate of 13.7%, intention-to-treat analysis applied.
Kinkead et al., 2017 [52]	**Variables**: Fatigue, QoL. **Instruments**: MFI, PROMIS, Q-LES-Q. **Assessment**: Baseline, week 3, and week 6.	**Therapy**: Swedish massage vs. Light touch vs. Waitlist. **Sessions**: 6 sessions. **Duration**: 45 min weekly.	The IG achieved a clinically significant reduction in fatigue and significant improvement in QoL compared to the CG. Context: N = 66 randomized; single-masked trial, credibility and expectancy biases did not account for the positive results.
Larkey et al., 2014 [40]	**Variables**: Fatigue, sleep quality, depression. **Instruments**: FSI, PSQI, BDI. **Assessment**: Baseline, 12 weeks, and 3 months post-intervention.	**Therapy**: Qigong/Tai Chi Easy vs. Sham Qigong. **Sessions**: 14 group sessions. **Duration**: 60 min weekly + 30 min/day home practice.	The IG achieved significant reduction in fatigue compared to sham, but no significant between-group difference for sleep or depression. Context: N = 101 randomized; 13.9% attrition, double-blind RCT using Sham Qigong as control.
Mao et al., 2024 [28]	**Variables**: Fatigue, sleep, anxiety, depression, stress, pain, QoL. **Instruments**: BFI, PSQI, HADS, PSS-10, BPI, FACT-B. **Assessment**: Baseline, 3, 6, 12, and 18 weeks.	**Therapy**: Infrared Laser Moxibustion (ILM) vs. Sham vs. Waitlist. **Sessions**: 12 sessions. **Duration**: 20 min, twice weekly for 6 weeks.	ILM reduced fatigue and improved sleep compared to waitlist; significant reductions appeared at 18 weeks compared to sham (delayed effect). Context: N = 140 randomized; intention-to-treat analysis, no serious adverse events.
Miranda et al., 2020 [32]	**Variables**: Spiritual distress, anxiety, depression, coping. **Instruments**: HADS, salivary amylase, Spiritual Distress Scale, RCOPE. **Assessment**: Pre, week 3 of RT, and last RT session.	**Therapy**: Intercessory prayer vs. Control. **Sessions**: Daily during RT. **Duration**: 1 h during radiotherapy.	The IG showed significant reduction in spiritual distress and negative religious coping; no significant difference for anxiety or depression. Context: N = 31 randomized; double-blind, salivary amylase differed at baseline.
Myers et al., 2019 [41]	**Variables**: Fatigue, cognition, sleep, distress, QoL. **Instruments**: BFI, FACT-Cog, PROMIS, RAVLT, Trail Making, MDASI. **Assessment**: Baseline, 8, and 12 weeks.	**Therapy**: Qigong vs. Gentle exercise vs. Survivorship support. **Sessions**: 8 group sessions. **Duration**: 60 min weekly + 15 min twice/day home practice.	The IG showed significant reduction in distress, improved self-reported cognitive function, and improved Trail Making A scores. Context: N = 50 randomized; high attrition (50% in gentle exercise group), low home practice adherence.
Nguyen et al., 2018 [42]	**Variables**: Quality of life. **Instruments**: FACT-B, TOI. **Assessment**: Baseline, week 3, end of EBRT, and 1 month post.	**Therapy**: Human Universal Energy (HUE) vs. Sham HUE. **Sessions**: Twice daily. **Duration**: 5 min each.	Limited to weak evidence of difference between HUE and sham for HRQoL. Context: N = 32 randomized; results heavily skewed by outliers, ANCOVA models were unstable, larger samples recommended.
Pelzer & Tröger, 2018 [57]	**Variables**: Safety, QoL, physical symptoms. **Instruments**: EORTC QLQ-C30, thermometry, neutrophil count. **Assessment**: 18 weeks and 5-year follow-up.	**Therapy**: Mistletoe injections vs. Control. **Sessions**: 54 sessions. **Duration**: 3/week for 18 weeks.	The IG showed significant improvements in role and emotional functioning, and reduced pain, appetite loss, and diarrhea. Context: N = 95 randomized; open-label trial due to expected local skin reactions from mistletoe.
Serra et al., 2023 [53]	**Variables**: Hot flushes, QoL. **Instruments**: MenQol scale, HF diary. **Assessment**: Week 0, post-treatment, and 1-month follow-up.	**Therapy**: Therapeutic acupuncture vs. Sham acupuncture. **Sessions**: 14 sessions. **Duration**: Twice weekly for 5 weeks, then once weekly for 4 weeks.	Statistically significant drop in hot flash severity compared to sham; no significant placebo response observed. Context: N = 54 enrolled, 47 completed; Streitberger placebo device used for sham control.
Shao et al., 2026 [48]	**Variables**: Cancer-related fatigue (CRF), sleep quality, and quality of life (QoL). **Instruments**: Piper Fatigue Scale (PFS), Pittsburgh Sleep Quality Index (PSQI), and the Functional Assessment of Cancer Therapy-Breast (FACT-B) questionnaire. **Assessment**: Baseline (T0), after 4 weeks of treatment (T1), and at 8 weeks (T2).	**Therapy**: Shenqi Fuzheng (SQFZ) traditional Chinese formula granules dissolved in warm water vs. Placebo (granules with identical appearance and taste but no active ingredients). **Duration**: Twice daily. 8 consecutive weeks during chemotherapy cycles.	The SQFZ group showed a significant reduction in fatigue scores (PFS) and improvement in sleep quality (PSQI) compared to the placebo group (*p* < 0.05). Additionally, a significant increase in overall quality of life (FACT-B) was observed. Context: Randomized double-blind placebo-controlled trial conducted in a clinical hospital setting.
Tambour et al., 2018 [43]	**Variables**: Arm volume, circumference, symptoms. **Instruments**: Water displacement, tape measure, VAS, EQ-5D-5L. **Assessment**: Baseline, 1 month, and 7 months.	**Therapy**: Manual Lymphatic Drainage (MLD) + CDT vs. CDT alone. **Sessions**: 8 sessions. **Duration**: 30 or 60 min, twice weekly for 4 weeks.	Adding MLD to standard care did not result in a significant extra reduction in arm volume or patient-reported symptoms. Context: N = 77 randomized; single-blind equivalence trial, MLD may not be a necessary component of CDT.
Tsai et al., 2021 [47]	**Variables**: Severity of nausea, vomiting, and retching; self-care for CINV. **Instruments**: Index of Nausea, Vomiting, and Retching (INVR) and the Rhodes Index of Nausea, Vomiting and Retching (translated version). **Assessment**: Baseline (Day 1 of chemotherapy), and daily follow-ups through Day 5 of the chemotherapy cycle.	**Therapy**: Acupressure at the Neiguan (PC6) point using an adjustable wristband with a plastic peg (Sea-Band) vs. Standard care (routine antiemetic medication only). **Session**: 5 consecutive days during the chemotherapy cycle. **Duration:** Pressing peg for 1–2 min when feeling nauseous.	The acupressure group showed significantly lower scores for nausea, vomiting, and retching severity (*p* < 0.05) compared to the control group. The intervention also significantly improved patients’ self-care ability to manage CINV. Context: Randomized controlled trial conducted in a medical center oncology ward.
Wang et al., 2024 [54]	**Variables**: Chronic pain, anxiety, depression, QoL. **Instruments**: BPI, HADS, QOLCSV-C, FACT-B. **Assessment**: Baseline, week 5, and week 9.	**Therapy**: Breathing exercise (slow pursed lip) vs. Waitlist. **Sessions**: Daily (for 4 weeks). **Duration**: Practice daily for 4 weeks.	The IG showed significant reductions in chronic pain, anxiety, depression, and improvements in QoL. Context: N = 72 randomized; concurrent pain medication and baseline expectations were identified as significant confounding factors.
Yun et al., 2017 [58]	**Variables**: Depression, anxiety, perceived stress, QoL, sleep, posttraumatic growth. **Instruments**: CES-D, BAI, PSS, FACT-B, SWLS, PTGI, PSQI. **Assessment**: Baseline, 4 weeks, and 8 weeks.	**Therapy**: Mind Subtraction Meditation (MSM) vs. Self-management education. **Sessions**: 16 sessions. **Duration**: 120 min, twice weekly for 8 weeks.	The IG showed significantly reduced depression, anxiety, and perceived stress; and increased QoL, life satisfaction, posttraumatic growth, and sleep quality. Context: N = 52 randomized; high class attendance demonstrated clinical feasibility and acceptance.
Zhang et al., 2020 [62]	**Variables**: Sleep, fatigue, QoL, anxiety, depression, pain. **Instruments**: PSQI, BFI, FACT-B, ISI, Actiwatch, Sleep Diary, HADS, BPI. **Assessment**: Baseline to week 42.	**Therapy**: Electroacupuncture + auricular acupressure vs. Sham. **Sessions**: 18 sessions over 12 weeks. **Duration**: Over 18 weeks.	Active acupuncture significantly improved sleep onset, total sleep time, anxiety, depression, and QoL. Context: N = 138 randomized; active group had a significantly lower discontinuation rate than sham.
Zhu et al., 2018 [34]	**Variables**: Severity of fatigue and quality of sleep. **Instruments**: Multidimensional Fatigue Inventory (MFI) and the Pittsburgh Sleep Quality Index (PSQI). **Assessment**: Baseline (before chemotherapy), immediately after the 4-week intervention, and 1 month after the intervention ended.	**Therapy**: Foot reflexology (massaging specific points on the foot related to the breast, chest, and lymphatic system) vs. Control (routine care). **Sessions**: 4 consecutive weeks (8 sessions total). **Duration:** Each session lasting 30 min (15 min per foot). 2 sessions per week.	The reflexology group showed a significant reduction in fatigue scores (*p* < 0.001) and improvement in sleep quality (*p* < 0.001) compared to the control group immediately and one month post-intervention. The study concluded that foot reflexology is an effective nursing intervention to manage side effects in chemotherapy patients. Context: Randomized controlled trial conducted in a chemotherapy center.
Zick et al., 2016 [59]	**Variables**: Fatigue, sleep, QoL. **Instruments**: BFI, PSQI, LTQL. **Assessment**: Baseline, 3, 6, and 10 weeks.	**Therapy**: Self-acupressure (relaxing or stimulating) vs. Usual care. **Sessions**: Daily. **Duration**: 3 min per point daily for 6 weeks.	Both acupressure types significantly improved fatigue; only relaxing acupressure significantly improved sleep quality and QoL vs. usual care. Context: N = 288 randomized; intent-to-treat, normal fatigue achieved in 66.2% (relaxing) and 60.9% (stimulating).
The Authors.			

IG: intervention group; PG: placebo group; CG: control group.

**Table 3:** Summary of potential benefits by complementary therapy category.

Therapy Category	Specific Interventions	Positive Effects	Mixed/No Significant Effects
Acupuncture & Acupressure	Manual/Electroacupuncture, Acupressure, Laser Moxibustion	Pain, Fatigue, Sleep, CINV, Hot Flashes	Lymphedema (arm volume reduction)
Mind–Body Practices	Mindfulness, Yoga, Qigong, Tai Chi, Meditation, Breathing	Stress, Anxiety, Depression, Fatigue, Sleep	Fatigue (Yoga mixed), Nausea (Yoga)
Massage & Manual Therapies	Classical/Swedish Massage, Neuromuscular Taping	Pain, Fatigue, Neuropathy symptoms, QoL	Lymphedema (Manual Lymphatic Drainage)
Music & Dance Therapy	Music intervention, Dance–movement therapy	Anxiety, Pain, Stress, QoL	Fatigue (Music/Dance mixed)
Natural Products & Homeopathy	Peppermint, Ginseng, Mistletoe, Homeopathic products	CINV, QoL, Attention/Cognition, Fatigue	Anorexia
The Authors.			

## Data Availability

No new data were created or analyzed in this study.

## Supplementary Materials

The following supporting information can be downloaded at: https://www.mdpi.com/article/10.3390/healthcare14111588/s1. Figures S1 and S2: Risk-of-bias results using the RoB 2.0 tool; Table S1: PEDro scale results. File S1: PRISMA 2020 Checklist.
