# Supplementary material for: Potential Benefits of Complementary Therapies for Women with Breast Cancer Undergoing Oncological Treatment: A Systematic Review
Source: Healthcare (Basel). 2026 Jun 4;14(11):1588. doi: 10.3390/healthcare14111588 (PMC13256381; doi:10.3390/healthcare14111588)
Supplement: Supplementary file 1 [file healthcare-14-01588-s001.zip › healthcare-4288334-Table S1.pdf]

Table S1. PEDro scale results.

Author & Year	PEDro Scale Items											T.P.
	1	2	3	4	5	6	7	8	9	10	11	
Aguilar Vallim et al., 2019	Yes	Yes	No	Yes	No	No	No	Yes	No	Yes	Yes	5/10*
Anestin et al., 2017	Yes	Yes	No	Yes	No	No	No	Yes	Yes	Yes	Yes	7/10
Anestin et al., 2022	Yes	Yes	Yes	Yes	No	No	Yes	Yes	Yes	Yes	Yes	8/10
Bao et al., 2018	Yes	Yes	No	Yes	No	No	No	Yes	Yes	Yes	Yes	7/10
Bower et al., 2015	Yes	Yes	Yes	Yes	No	No	No	Yes	No	Yes	Yes	6/10
Brinkhaus et al., 2019	Yes	Yes	Yes	Yes	No	No	No	Yes	Yes	Yes	Yes	7/10
Chuang & Chen, 2014	Yes	Yes	Yes	Yes	No	No	Yes	Yes	Yes	Yes	Yes	8/10
Cohen et al., 2021	Yes	Yes	Yes	Yes	No	No	No	Yes	Yes	Yes	Yes	7/10
Conejo et al., 2018	Yes	Yes	Yes	Yes	Yes	No	Yes	Yes	No	Yes	Yes	8/10
Dolev et al., 2021	Yes	Yes	Yes	Yes	Yes	Yes	Yes	Yes	No	Yes	Yes	9/10
Eng et al., 2025	Yes	Yes	Yes	Yes	Yes	Yes	Yes	No	No	Yes	Yes	8/10
Hamidian et al., 2023	Yes	Yes	Yes	Yes	Yes	Yes	Yes	Yes	No	Yes	Yes	9/10
Ho et al., 2016	Yes	Yes	Yes	Yes	Yes	No	No	Yes	Yes	Yes	Yes	8/10
Hsieh et al., 2019	Yes	Yes	Yes	Yes	Yes	No	Yes	No	Yes	Yes	Yes	8/10
Izgu et al., 2019	Yes	Yes	Yes	Yes	No	No	Yes	Yes	Yes	Yes	Yes	8/10
Jafarimanesh et al., 2020	Yes	Yes	Yes	Yes	Yes	Yes	Yes	Yes	Yes	Yes	Yes	10/10
Jung et al., 2025	Yes	Yes	Yes	Yes	Yes	No	Yes	Yes	Yes	Yes	Yes	9/10
Khosropanah et al., 2023	Yes	Yes	No	Yes	No	No	No	Yes	No	Yes	Yes	5/10*
Kinkead et al., 2017	Yes	Yes	Yes	Yes	No	No	No	Yes	Yes	Yes	Yes	7/10
Larkey et al., 2014	Yes	Yes	Yes	Yes	Yes	No	Yes	Yes	No	Yes	Yes	8/10
Mao et al., 2024	Yes	Yes	Yes	Yes	Yes	Yes	Yes	Yes	Yes	Yes	Yes	10/10
Miranda et al., 2020	Yes	Yes	Yes	Yes	Yes	No	Yes	Yes	Yes	Yes	Yes	9/10
Myers et al., 2019	Yes	Yes	Yes	Yes	No	No	Yes	Yes	Yes	Yes	Yes	8/10
Nguyen et al., 2018	Yes	Yes	Yes	Yes	Yes	No	No	Yes	Yes	Yes	Yes	8/10
Pelzer & Tröger, 2018	Yes	Yes	Yes	Yes	No	No	No	Yes	No	Yes	Yes	6/10

Rostami et al., 2026	Yes	Yes	Yes	Yes	Yes	No	Yes	Yes	Yes	Yes	Yes	5/10*
Serra et al., 2023	Yes	Yes	Yes	Yes	Yes	No	No	Yes	No	Yes	Yes	7/10
Shao et al., 2026	Yes	Yes	Yes	Yes	No	No	Yes	Yes	Yes	Yes	Yes	8/10
Tambour et al., 2018	Yes	Yes	Yes	Yes	No	No	Yes	Yes	Yes	Yes	Yes	8/10
Tsai et al., 2021	Yes	Yes	Yes	Yes	No	No	Yes	Yes	Yes	Yes	Yes	8/10
Wang et al., 2024	Yes	Yes	Yes	Yes	No	No	No	Yes	Yes	Yes	Yes	7/10
Yun et al., 2017	Yes	Yes	No	Yes	No	No	No	Yes	Yes	Yes	Yes	6/10
Zhang et al., 2020	Yes	Yes	Yes	Yes	Yes	No	Yes	Yes	No	Yes	Yes	8/10
Zhu et al., 2018	Yes	Yes	Yes	Yes	Yes	No	Yes	Yes	Yes	Yes	Yes	9/10
Zick et al., 2016	Yes	Yes	Yes	Yes	No	No	No	No	Yes	Yes	Yes	6/10

T.P.: Total Puntuation. * Studies excluded because they scored less than 6 on the PEDro Scale
