# Supplementary figures and images for: Potential Benefits of Complementary Therapies for Women with Breast Cancer Undergoing Oncological Treatment: A Systematic Review
Source: Healthcare (Basel). 2026 Jun 4;14(11):1588. doi: 10.3390/healthcare14111588 (PMC13256381; doi:10.3390/healthcare14111588)

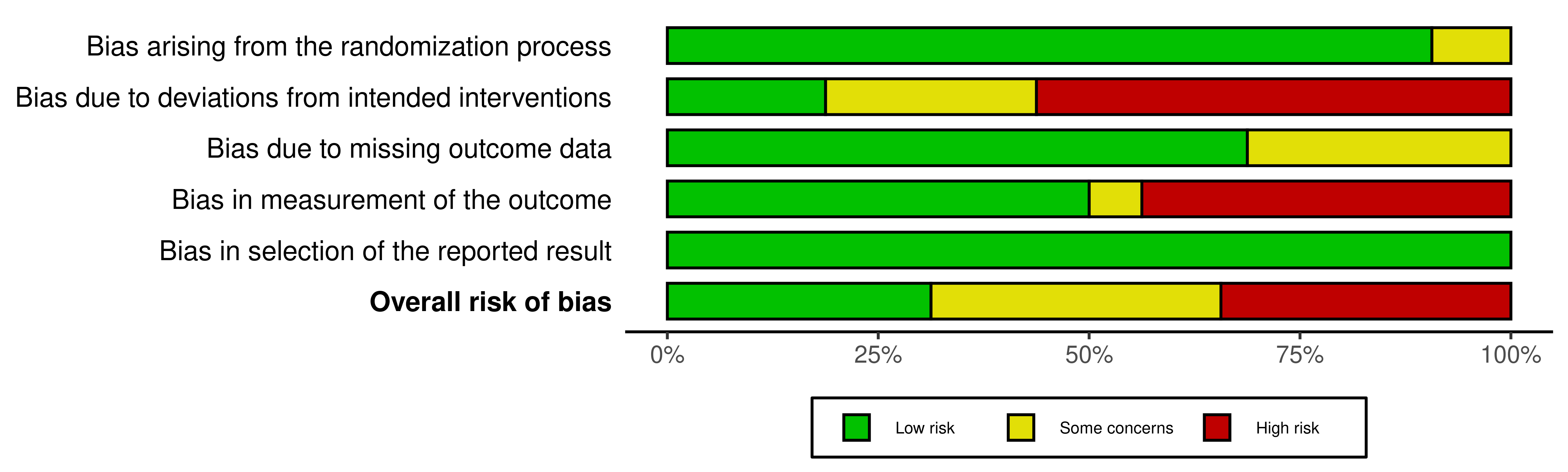

Supplement: Supplementary file 1 [file healthcare-14-01588-s001.zip › healthcare-4288334-Figure S2.tiff]
